# Chinese ideological and political teachers’ job satisfaction, loving pedagogy, and their professional success

**DOI:** 10.3389/fpsyg.2022.976397

**Published:** 2022-11-17

**Authors:** Yi Li, Xinpeng Wang

**Affiliations:** ^1^School of Marxism, Changshu Institute of Technology, Suzhou, China; ^2^School of Foreign Languages, Changshu Institute of Technology, Suzhou, China

**Keywords:** job satisfaction, loving pedagogy, professional success, IAP teachers, positive psychology

## Abstract

Job satisfaction, loving pedagogy, and teachers’ professional success, as three major emotional-psychological variables, have gradually gained its momentum in educational settings. The relationship among them has remained unknown. To address the existing gap, the current study set out to investigate the relationship among Chinese Ideological and Political (IAP) teachers’ job satisfaction, loving pedagogy, and their professional success. This non-experimental study employed three questionnaires adopted from and validated by prior studies. To carry out the study, an electronic survey created through *Wenjuanxing*, a computer program for conducting an online survey in China, was employed and convenience sampling technique was used. One thousand one hundred and eighty-nine Chinese IAP teachers with different majors, various academic degrees, and diversified teaching experiences voluntarily took part in the present study. With statistical analysis carried out by AMOS 23.0, the results of the study showed that job satisfaction and loving pedagogy could jointly predict 46.8 of the variance of teachers’ professional success. Both variables were the significant predictors of professional success, while loving pedagogy was a better predictor, solely explaining 39.4 of teacher success’s variance. Based on the findings, some pedagogical implications for educational institutions, administrators, and IAP teachers were discussed in the article. Future research directions and limitations were also mentioned.

## Introduction

The foundation of a society’s prosperity and development is inherently related to the *status quo* of its educational settings and organizations. Researchers on educational issues believe that among all the elements influencing the performances of an educational institution, teachers are the most deciding factor in the context of education (Acton and Glasgow, 2015; Han and Wang, 2021). Teachers are in a position of bringing upon inspirations and changes in students by practicing loving pedagogy and manipulating a multitude of variables (Mellati et al., 2013; Lindqvist et al., 2014). There is no doubt that teaching is regarded as a stressful and burdensome occupation and job satisfaction is not easy to attain among teachers (Richards et al., 2016). Nevertheless, according to the previous studies, teachers, who firmly implements loving pedagogy and displays a higher level of job satisfaction, have more job opportunities and better professional developments (Puni et al., 2018; Kangas-Dick and O’Shaughnessy, 2020). Those teachers practicing loving pedagogy and enjoying a high level of job satisfaction are bound to exert long-lasting effect upon learners, educational institutions, and administrators alike.

Despite the undeniable role of loving pedagogy and job satisfaction in teachers’ professional success (MacIntyre et al., 2016; Wang et al., 2022), the prediction of these two profession-related factors on Chinese IAP teachers’ success have not been widely studied (Rezaee et al., 2018; Baluyos et al., 2019). Furthermore, no original study has assessed the correlation of these three constructs at the same time or determined the better predictor of teachers’ success. The current study seeks to minimize the gaps by determining the joint prediction of Chinese IAP teachers’ professional success by loving pedagogy and job satisfaction. This article starts with the empirical literature related to teachers’ job satisfaction, loving pedagogy, and their professional success. Next is an analysis of an empirical study on the correlation among Chinese IAP teachers’ job satisfaction, loving pedagogy, and their professional success. Finally, limitations and future research directions are suggested.

## Review of literature

### Job satisfaction

Teachers’ job satisfaction pertains to their affective reactions to the teaching career and their professional role (Jiang et al., 2019). As Malinen and Savolainen (2016) pinpointed, job satisfaction is subjective to a variety of internal and external factors that are called “*work content factors*” and “*work context factors*,” respectively. They stated that one’s job satisfaction is determined by the inherent features of the profession (work content factors) and the workplace conditions (work context factors). Accordingly, besides the nature of the teaching profession, teachers’ job satisfaction may be influenced by some contextual factors, including class size, classroom facilities, and colleagues’ behaviors (Lopes and Oliveira, 2020). To date, some researchers have evaluated the role of job satisfaction in educational environments by investigating its potential effects on teacher commitment (Bashir and Gani, 2020), teacher cooperation (Olsen and Huang, 2019), teacher professional performance (Baluyos et al., 2019), and teacher success (Rezaee et al., 2018). It serves to enhance a teacher’s performance as well as individualized feeling of professional success. It demonstrates IAP teachers’ favorable attitude toward their educational institutions, students as well as daily teaching practices. Diversified definitions for the construct of job satisfaction, such as fulfillment and job contentment, have been put forward by researchers starting from differentiated perspectives (Capecchi and Piccolo, 2016; Abate et al., 2018; Berliana et al., 2018). Job satisfaction generally reflects how much individuals like their jobs. Researchers believe that job satisfaction covers a series of personalized attitudes toward several dimensions of the job such as the work itself, educational setting, salary, supervisors, and colleagues (Mellati et al., 2018). Job satisfaction does not arise from a single element at a specific time but a combination of several factors throughout the educational process (Fathi et al., 2020; Dreer, 2021). Dissatisfaction toward those factors might result in reduced job commitment, ever-lasting complaints, poor efficiency, and even early retirement (Lee, 2017; Lee and Shin, 2017; Alavi et al., 2021). Job satisfaction is not only related to the material side of a profession but also due to its psychological and social factors. A person might feel satisfied if there is no gap between what one desires and the perception of reality. Undoubtedly, a satisfied, loving teacher inspires a learner to pursue further while a dissatisfied one is unable to motivate a learner due to favorable or unfavorable organizational pattern. Further, bi-directional relationship between job satisfaction and work behavior is also emphasized as job satisfaction serves as a determinant of work behavior and as determined by work behavior (Ziegler and Schlett, 2016). While a multitude of previous studies have been conducted on job satisfaction, further research is needed to expound on the intriguing interaction among job satisfaction, loving pedagogy and IAP teachers’ professional development due to the characteristics and intricate nature of IAP teachers.

### Loving pedagogy

Teaching, learning, and even researching are intertwined with one’s affect, emotion, and attitude. With the introduction of Positive Psychology (PP), this affective turn in education starts to shift its attention from negative elements to positive emotions with loving pedagogy as one of them (Dewaele et al., 2019; Dewaele and Li, 2020; Wang et al., 2021). Additionally, they are required to be proficient in employing interpersonal communication skills to offer a lovely learning atmosphere (Xie and Derakhshan, 2021; Derakhshan, 2022). In the educational setting, love has the inherent power to motivate learners to pursue knowledge, display their potential, and achieve their goals. Love manifests itself through a caring environment, mutual rapport between the teacher and students, and classroom practices (Zhao and Li, 2021). Hierarchy of human needs proposed by Maslow (1954) pinpointed the precedence of love over others with physiological and safety needs at the bottom followed by love and belonging, esteem, and self-actualization at the upper ladder. Loving and feeling loved have always been very important aspects in our lives. Nevertheless, love is deemed divine and holy in the religious sense, and has long been viewed as a taboo stepping over the boundary in the academia due to its sexual implication and ethical consideration (Cousins, 2017). The combination of love and pedagogy has only recently gained its momentum. Loving pedagogy, a recent termed developed by various researchers (Loreman, 2011; Yin et al., 2019; Wang et al., 2021), refers to teachers’ sensitivity and support toward individual learners’ emotional needs and academic engagement, which includes nine main elements of *passion*, *kindness*, *empathy*, *intimacy*, *bonding*, *sacrifice*, *forgiveness*, *acceptance*, and *community*. Simply, Teaching and learning are acts of love and successful instruction can only be achieved in a loving setting. Research shows that a loving pedagogy place learners at the center of their practice, fit within professional practice, and enrich experiences for children and educators alike (Grimmer, 2021). Using love as pedagogy serves as an antidote to superficial learning and absence of love in education renders teaching a simple training.

### Teacher success

Teacher success plays a pivotal role in the effective daily operations among learners and educational institutions (Pishghadam et al., 2021). Teachers’ professional success, without a shadow of a doubt, has an enormous impact on learners’ academic outcomes (Gershenson, 2016), classroom engagement (Franklin and Harrington, 2019), and their willingness to attend classes (Pishghadam et al., 2019). According to previous research, learners who view their teachers as successful tend to attend classes and participate in academic activities. Owing to the value of teachers’ professional success, factors supporting teachers to succeed in their profession need to be recognized. To address this necessity, many researchers have evaluated the role of personal qualities, including autonomy, self-efficacy, emotional intelligence, and creativity in teachers’ professional success (e.g., Sezgin and Erdogan, 2015; Chen, 2019; Martin and Mulvihill, 2019; Derakhshan et al., 2020, among others). In the ideological and political course, teachers vigorously promote the cultivation of positive psychological quality and self-efficacy among learners. A further exploration of teacher- and learner-related factors contributing to teacher success is needed in order to verify its application in the domain of ideological and political education. Vigorous empirical studies *via* questionnaires have been tried to confirm the construct of teacher success with the following factors such as teacher creativity, professional identity, grit, and optimistic explanatory style expounded on more thoroughly (Pishghadam et al., 2012; Derakhshan et al., 2020). Considering the complex and challenging nature of IAP teaching task, teacher success cannot be achieved in all circumstances; thus, it is vital to formulate and confirm factors boosting or obstructing teacher success (Pishghadam et al., 2021). Studies show that little is known concerning the relationship among job satisfaction, loving pedagogy, and teacher success. Accordingly, the present study address the following research questions.

### Research questions

(1) Are there any correlations between Chinese IAP teachers’ job satisfaction, loving pedagogy, and their professional success?
(2) Do job satisfaction and loving pedagogy predict Chinese IAP teachers’ professional success?

## Materials and methods

### Research design

The current study is a non-experimental study by employing three kinds of questionnaires adopted from prior studies, which took the teacher population as the sample and have validated those questionnaires: the Job Satisfaction Scale (JSS), developed by Feng (2007), Dispositions toward Loving Pedagogy Scale (LPS) developed by Yin et al. (2019), and Successful Teachers Scale (STS), developed by Wang and Zhou (2019). Also, items in the three online questionnaires were translated into Chinese, the native language of the participants, so that they could understand them well. The sampling technique employed in this study is convenience sampling as it is not feasible to perform other types of on-site sampling due to the impact of COVID-19. Statistical analyses in the current study were implemented using AMOS 23.0.

### Participants

In this study, a sample of 1,189 IAP teachers took part in completing the questionnaires on job satisfaction, loving pedagogy, and professional success. To enhance generalizability of the findings of this study to the wider population of IAP teachers across China, they were selected from 30 diverse Chinese provinces, autonomous regions and municipalities including Jiangsu (*N* = 1100), Shanghai (*N* = 49), Zhejiang (*N* = 19), and Henan (*N* = 7), etc. Besides, many of the participants major in Ideological and Moral Cultivation and Legal Basis (*N* = 208), Fundamentals of Marxism (*N* = 52), Introduction to Mao Zedong Thought and Theoretical System of Socialism with Chinese Characteristics (*N* = 328), Outline of Modern Chinese History (*N* = 26), An Introduction to Xi Jinping Thought on Socialism with Chinese Characteristics for a New Era (*N* = 23), Situation and Policy (*N* = 88), Military Theory (*N* = 9), Party History and Party Building (*N* = 7), and other (*N* = 448), etc. (see Table 1). The participants were teaching in colleges and universities with a range of age from around 20 to over 60, various degrees from associate to Ph.D., and diversified length of teaching experiences ranging from within 5 years to over 25 years. Informed consent was obtained from all the participants. The demographic information of the participants is presented in Table 1.

**TABLE 1:** Demographic information of the participants.

Variable	Frequency
Gender	Male = 164
	Female = 1,025
Major	Ideological and moral cultivation and legal basis = 208
	Fundamentals of Marxism = 52
	Introduction to Mao Zedong thought and theoretical system of socialism with Chinese characteristics = 328
	Outline of modern Chinese history = 26
	An introduction to Xi Jinping thought on socialism with Chinese characteristics for a new era = 23
	Situation and policy = 88
	Military theory = 9
	Party history and party building = 7
	Other = 448
Academic degree	Associate of Arts = 755
	Bachelor of Arts = 72
	Master of Arts = 192
	Ph.D. = 119
	Other = 51
Teaching experience	(1–5) = 688
	(6–10) = 63
	(11–15) = 93
	(16–20) = 70
	(21–25) = 39
	More than 25 = 236

### Instruments

Three questionnaires were employed in this study to gather the data, respectively concerning job satisfaction, loving pedagogy, and professional success of IAP teachers across China.

#### Job satisfaction scale

To measure IAP teachers’ satisfaction, the Job Satisfaction Scale (JSS), developed by Feng (2007), was used. The scale compromises 26 items with a 5-point Likert scale, ranging from 5 (strongly agree) to 1 (strongly disagree); thus, the participants may obtain a score between 26 and 130. The JSS includes five factors, including self-fulfillment (items 1, 2, 3, 4, 5, 6, 7), work intensity (items 8, 9, 10, 11, 12), salary income (items 13, 14, 15, 16, 17), leadership relations (items 18, 19, 20, 21, 22), and collegial relations (items 23, 24, 25, 26).

#### Loving pedagogy scale

In the present study, IAP teachers’ loving pedagogy was measured with the Dispositions toward Loving Pedagogy Scale (LPS) developed by Yin et al. (2019). The scale compromises 29 items, rated on a five-point Likert scale ranging from 5 (strongly agree) to 1 (strongly disagree); thus, the participants may obtain a score between 29 and 145, on five factors, concerning acceptance of diversity and classroom community (items 1, 2, 3, 4, 5, 6, 7, 8, 9), intimacy (items 10, 11, 12, 13, 14, 15), bonding and sacrifice (items 16, 17, 18, 19, 20, 21, 22), empathy (items 23, 24, 25), forgiveness and kindness (items 26, 27, 28, 29).

#### Successful teachers scale

The STS, developed by Wang and Zhou (2019), comprises 6 factors in the form of 25, rated on 5-point Likert-type items ranging from 5 (strongly agree) to 1 (strongly disagree); thus, the participants may obtain a score between 25 and 125. The STQ covers six factors, including familiarity with Marxism and moral cultivation (items 1, 2, 3, 4), teaching booster (items 5, 6, 7, 8, 9, 10), learning booster (items 11, 12, 13, 14, 15), research involvement (items 16, 17, 18, 19), teacher-student relationship (items 20, 21, 22), and organizational contribution (items 23, 24, 25).

### Data collection procedure

Initially, to collect data, an electronic survey form containing three scales of JSS, LPS, and STS, was created through *Wenjuanxing*, a computer program for conducting an online survey in China to help researchers collect data from different regions across China, shared through the WeChat application and the QQ application. In effect, the questionnaires were administered and completed online. The Chinese participants scanned a QR code or clicked the URL to complete the questionnaire, which began on February 18th, 2022 and lasted until the end of March, 2022.

Although there was no time limit for submission of the responses, the teachers took 15 min to complete the questionnaire on average. Given that the present study included participants from various regions across China with the COVID-19 lingering on, using electronic questionnaires was preferred as a convenient and safe way of data collection for the researchers and participants alike. Further, all the items were translated into Chinese, the native language of the participants, to increase user-friendliness to the study. The participants were all fully notified of their rights to withdrawal from the study at any time for any discomfort. When they submitted the data, they were instructed how to give validated honest answers and were assured that their personal information would be anonymously used for research purpose only.

All the necessary instructions for completing the questionnaires were included in the online survey. The data collection process lasted for almost one and a half month and 1,189 electronic forms were completed with no missing data. After scrutinizing the collected responses for potential errors, the statistical analysis based on Amos software (version 23) was conducted for the exploration of answers to the research questions.

### Data analysis

#### Pre-processing of the data

Before starting data analysis, pre-processing of the data was implemented to exclude the inappropriate data. Originally, 1,189 answers were collected with no missing ones. Based on the standard deviation and average score of respondents’ answers, the data was inspected for certain patterns. Also, there are some reverse questions in the questionnaire, and the engagement of the respondents is predicted by the answers to the reverse questions. Consequently, cases with constant pattern (whether it is the highest score, the lowest score or the middle one) were identified and double checked, and 397 cases were excluded for unengagement. Thus, as a result of data screening and cleaning, 792 cases were retained for the main data analysis.

#### Construct validity

To confirm the construct validity, a CFA was carried out. The initial CFA model had three constructs (Job Satisfaction, Loving Pedagogy, and Teacher Success), each with items in second order. Each of the three constructs was carefully scrutinized for non-significant loadings in unstandardized estimation and/or low estimates (below 0.5) in standardized estimation. As is shown in Table 2, there were no non-significant unstandardized estimates. Yet, three items from job satisfaction, i.e., items 15, 16, and 26, and one item from loving pedagogy, i.e., item 27, had standardized estimates below 0.50. Both authors, along with some experts, re-evaluated those items and found that there are certain repetition between those items and other items. Thus, those items were discarded from further processing.

**TABLE 2:** Unstandardized and standardized estimates of the initial CFA model.

			Unstandardized				Standardized
			Estimate	S.E.	C.R.	*P*	Estimate
S01	<_–––_	SF	0.844	0.028	30.579	***	0.814
S02	<_–––_	SF	0.954	0.032	30.049	***	0.806
S03	<_–––_	SF	0.882	0.027	32.439	***	0.839
S04	<_–––_	SF	0.714	0.026	27.387	***	0.765
S05	<_–––_	SF	1.000				0.889
S06	<_–––_	SF	1.028	0.029	35.028	***	0.871
S07	<_–––_	SF	0.985	0.036	27.432	***	0.766
S08	<_–––_	WI	1.449	0.104	13.966	***	0.718
S09	<_–––_	WI	1.116	0.087	12.762	***	0.612
S10	<_–––_	WI	1.580	0.103	15.402	***	0.897
S11	<_–––_	WI	1.556	0.102	15.261	***	0.871
S12	<_–––_	WI	1.000				0.521
S13	<_–––_	SI	1.425	0.069	20.689	***	0.861
S14	<_–––_	SI	1.456	0.069	20.983	***	0.903
S15	<_–––_	SI	0.519	0.056	9.270	***	0.354
S16	<_–––_	SI	0.506	0.057	8.859	***	0.338
S17	<_–––_	SI	1.000				0.673
S18	<_–––_	LR	1.575	0.118	13.329	***	0.691
S19	<_–––_	LR	1.426	0.111	12.872	***	0.649
S20	<_–––_	LR	1.150	0.093	12.355	***	0.606
S21	<_–––_	LR	1.635	0.123	13.318	***	0.690
S22	<_–––_	LR	1.000				0.533
S23	<_–––_	CR	0.820	0.057	14.477	***	0.512
S24	<_–––_	CR	1.187	0.045	26.403	***	0.904
S25	<_–––_	CR	1.000				0.828
S26	<_–––_	CR	0.687	0.055	12.431	***	0.447
L01	<_–––_	DC	1.000				0.568
L02	<_–––_	DC	1.030	0.069	14.942	***	0.676
L03	<_–––_	DC	1.229	0.074	16.656	***	0.806
L04	<_–––_	DC	1.206	0.072	16.764	***	0.815
L05	<_–––_	DC	1.161	0.082	14.191	***	0.627
L06	<_–––_	DC	1.222	0.071	17.181	***	0.852
L07	<_–––_	DC	1.311	0.077	17.069	***	0.842
L08	<_–––_	DC	1.207	0.081	14.935	***	0.676
L09	<_–––_	DC	1.037	0.073	14.258	***	0.631
L10	<_–––_	IN	1.000				0.828
L11	<_–––_	IN	1.066	0.038	28.393	***	0.842
L12	<_–––_	IN	0.995	0.034	29.467	***	0.863
L13	<_–––_	IN	1.006	0.036	27.629	***	0.827
L14	<_–––_	IN	0.677	0.032	21.202	***	0.684
L15	<_–––_	IN	0.682	0.031	22.143	***	0.707
L16	<_–––_	BS	0.976	0.028	34.812	***	0.852
L17	<_–––_	BS	1.000				0.896
L18	<_–––_	BS	1.008	0.033	30.922	***	0.804
L19	<_–––_	BS	0.995	0.025	40.033	***	0.904
L20	<_–––_	BS	1.013	0.025	40.528	***	0.908
L21	<_–––_	BS	0.832	0.039	21.234	***	0.640
L22	<_–––_	BS	0.940	0.028	33.401	***	0.836
L23	<_–––_	EM	1.000				0.898
L24	<_–––_	EM	1.004	0.030	33.135	***	0.853
L25	<_–––_	EM	0.824	0.025	32.435	***	0.844
L26	<_–––_	FK	1.000				0.613
L27	<_–––_	FK	0.521	0.083	6.265	***	0.236
L28	<_–––_	FK	1.293	0.066	19.670	***	0.924
L29	<_–––_	FK	1.196	0.062	19.173	***	0.879
T01	<_–––_	MC	1.000				0.864
T02	<_–––_	MC	1.036	0.026	39.158	***	0.932
T03	<_–––_	MC	1.034	0.026	39.754	***	0.939
T04	<_–––_	MC	1.027	0.033	31.384	***	0.837
T05	<_–––_	TB	1.000				0.893
T06	<_–––_	TB	1.000	0.028	35.639	***	0.857
T07	<_–––_	TB	1.084	0.025	43.786	***	0.931
T08	<_–––_	TB	1.063	0.025	42.748	***	0.923
T09	<_–––_	TB	1.040	0.027	38.856	***	0.890
T10	<_–––_	TB	1.066	0.026	41.399	***	0.912
T11	<_–––_	LB	1.000				0.918
T12	<_–––_	LB	1.000	0.021	46.765	***	0.926
T13	<_–––_	LB	0.995	0.021	46.452	***	0.924
T14	<_–––_	LB	1.001	0.024	42.068	***	0.894
T15	<_–––_	LB	0.987	0.022	45.623	***	0.919
T16	<_–––_	RI	1.000				0.928
T17	<_–––_	RI	1.011	0.022	45.786	***	0.924
T18	<_–––_	RI	0.855	0.047	18.250	***	0.570
T19	<_–––_	RI	0.837	0.042	19.975	***	0.608
T20	<_–––_	TS	1.000				0.879
T21	<_–––_	TS	1.027	0.028	36.895	***	0.893
T22	<_–––_	TS	1.071	0.029	36.618	***	0.890
T23	<_–––_	OC	1.000				0.919
T24	<_–––_	OC	0.913	0.031	29.490	***	0.781
T25	<_–––_	OC	0.955	0.028	33.763	***	0.835

***Significant at 0.001.

**FIGURE 1:**
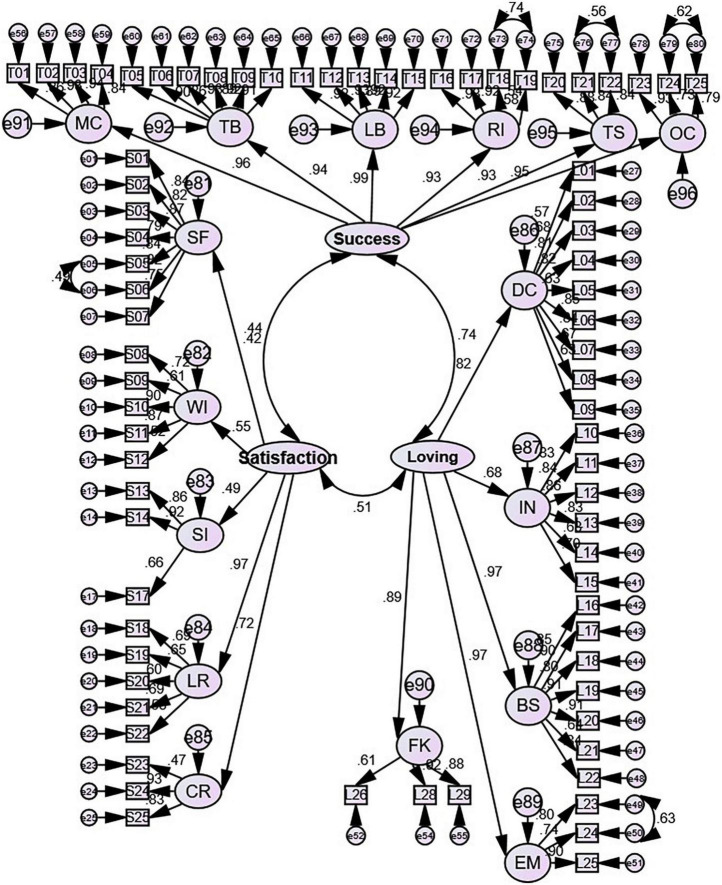
The final modified CFA model with standardized estimates.

After that, the modification indices with the threshold of 10 were checked, and modifications that were not contradictory to the literature were implemented. Figure 1 below demonstrates the final modified CFA model.

Table 3 below demonstrates the descriptive statistics for the component of the model. As shown in Table 3, all distributions of the scores showed normalcy as both skewness and kurtosis values were below the absolute value of 2.

**TABLE 3:** Descriptive statistics of the scores.

	*N*	Minimum	Maximum	Mean	SD	Skewness	Kurtosis
WI	792	1.00	5.00	3.0838	0.89168	–0.227	–0.054
SI	792	1.00	5.00	2.7761	0.88808	0.066	0.042
LR	792	1.60	5.00	3.7566	0.70349	–0.174	–0.335
CR	792	1.00	5.00	3.8855	0.70123	–0.018	–0.468
SF	792	1.00	5.00	4.5155	0.61573	–1.727	1.869
Satisfaction	792	1.74	5.00	3.7302	0.52245	–0.022	–0.114
DC	792	2.67	5.00	4.2765	0.60213	–0.506	–0.692
IN	792	1.33	5.00	4.0204	0.77059	–0.456	–0.256
BS	792	2.57	5.00	4.2522	0.63198	–0.400	–0.797
EM	792	1.67	5.00	4.1810	0.69438	–0.287	–0.855
FK	792	2.33	5.00	4.2908	0.62357	–0.498	–0.616
Loving	792	2.64	5.00	4.2068	0.57109	–0.310	–0.792
MC	792	2.75	5.00	4.5281	0.61308	–1.086	0.108
TB	792	1.83	5.00	4.4905	0.63600	–1.049	0.238
LB	792	1.00	5.00	4.0313	0.76334	–0.453	–0.187
RI	792	1.00	5.00	4.2494	0.71577	–0.684	0.091
TS	792	1.67	5.00	4.5063	0.62644	–1.085	0.435
OC	792	1.00	5.00	4.4007	0.68513	–0.948	0.382
Success	792	2.16	5.00	4.4486	0.60581	–0.944	–0.003

Next in Table 4 below, composite reliability (CR) and discriminant validity for each factor was calculated. As is shown in Table 4, each factor’s CR value is well above 0.7, revealing acceptable reliability. Further, the square root of average variance extracted (AVE) (the bold values in the table) was above intercorrelations of the factors, demonstrating discriminant validity (Fornell and Larcker, 1981). As reported in Table 4, there are significant correlations (values not in bold under Fornell-Larcker Criterion) between three pairs of factors. Strong correlation was confirmed between success and loving (*r* = 0.745) while moderate correlations were found between success and satisfaction (*r* = 0.438) as well as loving and satisfaction (*r* = 0.507).

**TABLE 4:** Composite reliability and discriminant validity of the factors.

	Fornell–Larcker criterion			
	C.R.	Satisfaction	Loving	Success
Satisfaction	0.968	**0.759**		
Loving	0.979	0.507***	**0.796**	
Success	0.988	0.438***	0.745***	**0.876**

***Correlation is significant at 0.001.

**FIGURE 2:**
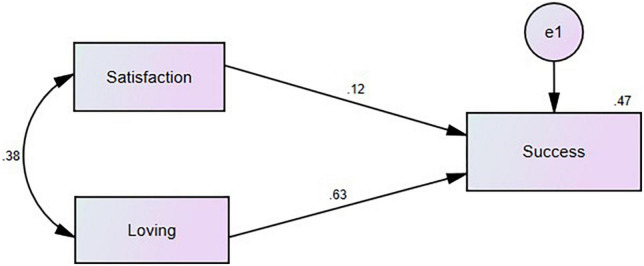
The final measurement model.

As correlation analysis above (see Table 4) demonstrates that teacher success has a significant correlation with both loving pedagogy and job satisfaction, a prediction model as in Figure 2 was set up to measure the predictability of teacher success by the two variables.

Table 5 below reports the results of the above analysis. By taking into account of the covariance between job satisfaction and loving pedagogy, the two variables could jointly predict 46.8 of the variance in teacher success. Both variables were significant predictors of teacher success, with loving pedagogy a better predictor (β = 0.628, *p* = 0.000 < 0.001), solely accounting for 39.4 of teacher success’s variance, and job satisfaction (β = 0.122, *p* = 0.000 < 0.001) explaining 1.5 of teacher success’s variance.

**TABLE 5:** Results of multiple linear regression analysis with SEM.

			Weight	S.E.	C.R.	*P*	β	*R* ^2^	Multiple correlation *R*^2^
Success	<_–––_	Loving	0.666	0.030	22.347	0.000	0.628	0.394	0.468
Success	<_–––_	Satisfaction	0.142	0.033	4.361	0.000	0.122	0.015	
Satisfaction	<_–_>	Loving	0.114	0.011	10.054	0.000	0.383		

## Discussion

The present study was to investigate the relationship among job satisfaction, loving pedagogy, and professional success. This study also intended to probe into job satisfaction and loving pedagogy’s joint prediction of Chinese IAP teachers’ professional success. In other words, the study set out to decide whether job satisfaction and loving pedagogy predict Chinese IAP teachers’ professional success.

As for the first research question, the findings of correlation analysis demonstrated that there was a significant, positive relationship among Chinese IAP teachers’ job satisfaction, loving pedagogy, and their professional success. Among them, the correlation between loving pedagogy and profession success is the strongest, followed by that between loving pedagogy and job satisfaction as well as that between job satisfaction and professional success. These findings were supported by the existent literature on those three notions. Prior studies confirmed that teachers with high level of job satisfaction and loving pedagogy are more likely to achieve profession success (Greenier et al., 2021). Consistent with Mousavi et al. (2012), the present study demonstrated that job satisfaction and loving pedagogy enable teachers to perform better and become more resilient to job-related burden and stress. Dreer (2021) has also proved the strong link between job satisfaction and their profession success. The outcome of this research concerning the strong relationship between job satisfaction and professional success is in line with that of Rezaee et al. (2018), who reported a close, direct link between job satisfaction and Iranian teachers’ success. This result also supports Baluyos et al. (2019) outcomes, which indicated that job satisfaction is tied to teachers’ occupational success. Besides, the result of this investigation regarding the favorable link between loving pedagogy and professional success corroborates the findings of Grimmer (2021), who found a remarkable association between loving pedagogy and teachers’ self-perceived success.

Satisfaction with the working environment could greatly enhance positive interrelationship in the workplace, which in turn boost their resilience to deal with potential problems in educational settings and achieve professional success in the end (Fathi et al., 2021). A dissatisfied teacher is contagious to the educational environment and will affect everyone’s productivity in educational settings. Administrators alike should keep this in mind so as to create a sense of satisfaction among teachers and remove its potential obstacles.

The combination of love and pedagogy, that is loving pedagogy, demonstrates teachers’ sensitivity and genuine support toward individual learners’ emotional needs and academic concerns. Based on the results of this study, loving pedagogy represents a much more significant correlation with teachers’ professional success than job satisfaction does. Put it simply, a loving pedagogy centers around individual learners, creates a loving educational setting, and renders teaching a process of loving and growth instead of simply an indifferent training. Thus, using love as pedagogy predicts teachers’ professional success as well as learners’ academic achievement.

As for the second research question, both job satisfaction and loving pedagogy significantly predict teachers’ professional success. Yet compared with job satisfaction, loving pedagogy is a much stronger predictor of teachers’ professional success. The pivotal role of loving pedagogy in predicting teachers’ professional success corroborates the findings of those researchers (Wilkinson and Kaukko, 2020; Zhao and Li, 2021) that implementing loving pedagogy in education setting enables teachers to outperform in their work, greatly draw learners’ attention, and boost their academic engagement. Apparently, teaching and learning are acts of love, and their success can only be achieved in a loving setting. Loving is no longer restricted to a religious, physiological sense, and its educational implications are worth further probing. In some sense, job satisfaction is an individual feeling, while loving pedagogy is always two-way and interactive, greatly predicting teachers’ professional success as well as learners’ academic gains.

## Conclusion and pedagogical implications

The findings of the present study showed that job satisfaction, loving pedagogy, and teachers’ professional success are significantly correlated with each other. Both job satisfaction and loving pedagogy predicted teachers’ professional success, among which loving pedagogy reported a much stronger prediction of Chinese IAP teachers’ professional success. Taken together, job satisfaction and loving pedagogy are two profession-related factors in boosting teachers’ success in Chinese IAP courses. The current investigation has gone some way toward raising teachers’ awareness of the value of love in educational settings. Teachers need to demonstrate due sensitivity and strong support toward learners’ emotional needs and academic concerns. Additionally, the present study also offers some implications for educational institutions and management. Considering that job satisfaction is also a predictor for Chinese IAP teachers’ professional success, school managers at various institutions are supposed to provide a better working environment to enhance their teachers’ job satisfaction. All in all, the emotional dimension of loving pedagogy and job satisfaction calls for great attention toward the emotional-psychological aspects of IAP teachers and individual learners in educational settings.

The current research is subject to several limitations. The first limitation lies in the fact that the present study focused on Chinese IAP teachers. Considering that the results of the investigation might not be applicable to other groups of teachers home and abroad, further studies concerning the emotional aspect of teachers might be implemented in similar or dissimilar educational contexts. The second limitation is that only close-ended scales were employed in the present study, which might be detrimental to the accuracy of the findings. To enhance the accuracy of the findings, some other data-gathering instruments such as open-ended questionnaires, structured and semi-structured interviews, and observation checklists could be employed in future studies. The third limitation of the present study was the ignorance of demographic data of the participants. It would be interesting to evaluate the mediating role of some demographic data such as gender, major, academic degree as well as teaching experience in the correlation of job satisfaction, loving pedagogy, and professional success.

## Data availability statement

The raw data supporting the conclusions of this article will be made available by the authors, without undue reservation.

## Ethics statement

The studies involving human participants were reviewed and approved by Academic and Ethics Committee of Changshu Institute of Technology. The patients/participants provided their written informed consent to participate in this study.

## Author contributions

YL was responsible for translating and distributing the questionnaire, collecting the data, and writing up the introduction, literature review, and research methodology. XW was responsible for conceptualizing the questionnaire, analyzing the data, and writing up the discussion and conclusion. Both authors made substantial and direct contribution to the current study and approved the submitted version.
